# Generation of CCR5-defective CD34 cells from ZFN-driven stop codon-integrated mesenchymal stem cell clones

**DOI:** 10.1186/s12929-015-0130-6

**Published:** 2015-03-26

**Authors:** Krissanapong Manotham, Supreecha Chattong, Anant Setpakdee

**Affiliations:** Department of Medicine, Molecular and Cell Biology Unit, Lerdsin General Hospital, Bangkok, Thailand

**Keywords:** Zinc finger nuclease, HIV, CCR5, Genome editing, Mesenchymal stem cells

## Abstract

**Backgrounds:**

Homozygous 32-bp deletion of the chemokine receptor 5 gene (*CCR5*) is associated with resistance to human immunodeficiency virus (HIV) infection, while heterozygosity delays HIV progression. Bone marrow transplantation (BMT) from a 32/32 donor has been shown to cure an HIV-infected patient. However, the rarity of this mutation and the safety risks associated with current BMT protocols are the major obstacles to this treatment. Zinc finger nuclease (ZFN) targeting is a powerful method for achieving genomic disruption at specific DNA sites of interest.

**Results:**

Taking advantage of the self-renewal and plasticity properties of stem cells, in this study, we successfully generated isogenic and six-cell clones of bone marrow-derived mesenchymal stem cells that carry the stop codon of the *CCR5* gene by using a ZFN-mediated homology-directed repair technique. These cells were expandable for more than 5 passages, and thus show potential to serve as an individual’s cell factory. When Oct4 was overexpressed, the mutated cells robustly converted to CD34+ progenitor cells.

**Conclusion:**

We here reported the novel approach on generation of patients own CD34 cells from high fidelity ZFN-mediated HDR MSC clones. We believe that our approach will be beneficial in future HIV treatment.

## Background

Chemokine receptor 5 (CCR5) functions as a co-receptor for human immunodeficiency virus (HIV) to enter into CD4 lymphocytes. Homozygosity of del32, a natural 32-bp deletion of the *CCR5* gene, confers strong resistance to HIV infection, while heterozygosity of this deletion results in a slower rate of HIV progression [1,2]. This finding suggested that bone marrow transplantation (BMT) from a del32 donor may be a beneficial HIV treatment method [1]. In 2009, Hütter and colleagues reported the results of a BMT from a del32/del32 donor to an HIV recipient [3]. Without any anti-retroviral drugs, the patient’s CD4 lymphocytes increased to normal levels and the virus remained undetectable for at least five years of follow-up [4,5]. These data provided strong evidence that HIV may be treatable, or at least be improved, by cell therapy. Nevertheless, allogeneic BMT for the treatment of HIV remains an impractical option. Since the frequency of del32 is low in the general population, and particularly in non-Caucasians [6,7], finding a suitable donor for each patient is not feasible. Moreover, the risks associated with the immunosuppressive regimens required following allogeneic BMT outweigh the risks associated with anti-HIV drugs.

Therefore, inactivation of *CCR5* by genetic manipulation of a patients’ own cells is a good alternative to avoid the drawbacks of donor shortage and immunosuppressive risks. Zinc finger nuclease (ZFN) targeting has recently been shown to be a promising method for disruption of genomic DNA at very specific loci [8-12]. ZFN is a hybrid protein consisting of an engineered DNA-binding zinc-finger, which attaches to non-specific nuclease, FokI. A pair of ZFNs is designed to specifically generate double-stranded breaks (DSBs) in genomic DNA between each binding site. Subsequently, the chromosomal DSBs initiate an error-prone repairing process known as non-homologous end-joining (NHEJ), which often results in an InDel mutation around the ZFN target site. Prezze’s and Holt’s research groups pioneered the use of ZFN-mediated InDel mutations in *CCR5* loci in CD4 lymphocyte and CD34 hematopoietic stem cells (HSCs), respectively [13,14]. Unfortunately, NHEJ is an imprecise process. InDel mutations are also unpredictable and are theoretically not equivalent to loss of function.

Apart from NHEJ, DSBs can also be repaired through a more precise mechanism known as homology-directed repair (HDR), which enables integration of a desirable, specific exogenous DNA sequence into the genome. Many groups have reported success of ZFN-mediated HDR in various human loci [10,15-17], including *CCR5* [18-20]. This approach is therefore a promising tool for mutation correction and site-specific gene insertion. Of particular interest, in highly proliferative cells, the use of ZFN homology base targeting was able to generate the expandable clones even from a single mutated cell [10,21]. A clone that carries the precise amount of an edited genome is ideal for cell therapy. Like drugs, the outcome as well as the toxicity of these high-fidelity clones is adjustable and predictable. Unfortunately, *in vitro* expansion of primary cell culture, including CD4 lymphocytes and HSCs, is limited; hence, obtaining an ideal, patient-specific edited clone population for therapeutic purposes has remained a challenge.

Somatic stem cells are post-natal stem cells that have very high self-renewal and differential capacity. Bone marrow-derived mesenchymal stem cells (MSCs) are well-established somatic stem cells that are easily obtained through simple bone marrow aspiration [22,23]. The proliferation rate of MSCs is much higher than that of CD4 lymphocytes and HSCs and may be the highest among all primary cell cultures. Previous work has also shown the feasibility of ZFN-mediated exogenous gene insertion into *CCR5* loci in MSCs [20]. Taken together, we speculated that it might be possible to generate and enrich ZFN-mediated *CCR5*-specific gene integration clones in MSCs.Recent progress in stem cell research has demonstrated that cell phenotypes are (re)programmable. In 2006, Takahashi and Yamanaka reported the successful reprogramming of somatic cells into pluripotent cells via induction with a set of transcription factors [24]. Therefore, there is great interest in the potential combination of cell reprogramming and ZFN-mediated gene editing [21,25-28]. Apart from cell reprogramming, direct conversion from one cell type to another is another interesting approach that may have an additional advantage of genetic stability [29]. Recently, fibroblasts were directly converted to CD45+ HSCs by overexpressing Oct4 and then maintaining the cells in defined media [30]. Collectively, these findings led us to reason that cell conversion could be a very powerful tool for future HIV treatment in conjunction with ZFN-mediated HDR. To support our speculation, we here report the generation of CD34^+^/CD45^+^ cells that originated from ZFN-mediated gene integration of MSCs.

## Methods

### MSCs isolation and culture

MSCs were isolated from leftover bone marrow aspiration samples, using a standard protocol [31]. The study protocol was approved by *the Ethic Committee of Lerdsin General Hospital*. The cells were cultured in Dulbecco’s modified Eagle’s medium (DMEM) (Gibco, Grand Island, NY, U.S.A.) supplemented with 10% platelet lysate (Thai Red Cross Society, Bangkok, Thailand) without antibiotics [32]. Cells between passage 3 and 5 were used in the study. For phenotypic characterization of MSCs, cells were harvested and washed in phosphate-buffered saline (PBS) buffer. Then, 10^6^ cells were resuspended in 100 μl PBS and incubated with fluorescein isothiocyanate-, Phycoerythrin-, or Phycoerythrin-Cy 7-conjugated monoclonal antibodies against CD34, CD45, HLA-DR, HLA-ABC, CD79a, CD29, CD33, CD44, CD73, CD90, and CD11b for 30 min at room temperature. All antibodies were obtained from BD Biosciences (San Diego, CA, USA). After incubation, excess antibodies were washed off by adding PBS followed by centrifugation at 2000 rpm for 5 min. Antibody-incubated cells were resuspended in 200 μl of FACS solution and flow cytometry was performed by using a FACS Calibur system (BD Biosciences). The data were analyzed with Cell Quest Pro software (BD Biosciences). Adipogenic and osteogenic differentiation were induced with Human Mesenchymal Stem Cell Functional Identification Kit (R&D system, Minneapolis, MN, USA).

### Immunohistochemistry and immunofluorescence analysis

For immunostaining, cells were cultured in 4-well chamber slides and fixed with −20°C methanol:acetone (1:1) for 10 min. The cells were then incubated with 3% H_2_O_2_ to quench endogenous peroxidases, as previously described [33]. Primary antibodies for osteocalcin and Oct4 (Cell signaling, Danvers, MA, USA) were applied in 1% bovine serum albumin with 0.1% Tween in PBS (dilution 1:500) for 1 h at room temperature, followed by incubation with biotinylated secondary antibodies for 30 min. Either avidin peroxidase or avidin-Alexa Fluor conjugates were applied at the final step. Images were captured with the Olympus BX51 and Olympus DP71 systems (Olympus; Tokyo, Japan).

### Generation of the donor plasmids

**Figure 1:**
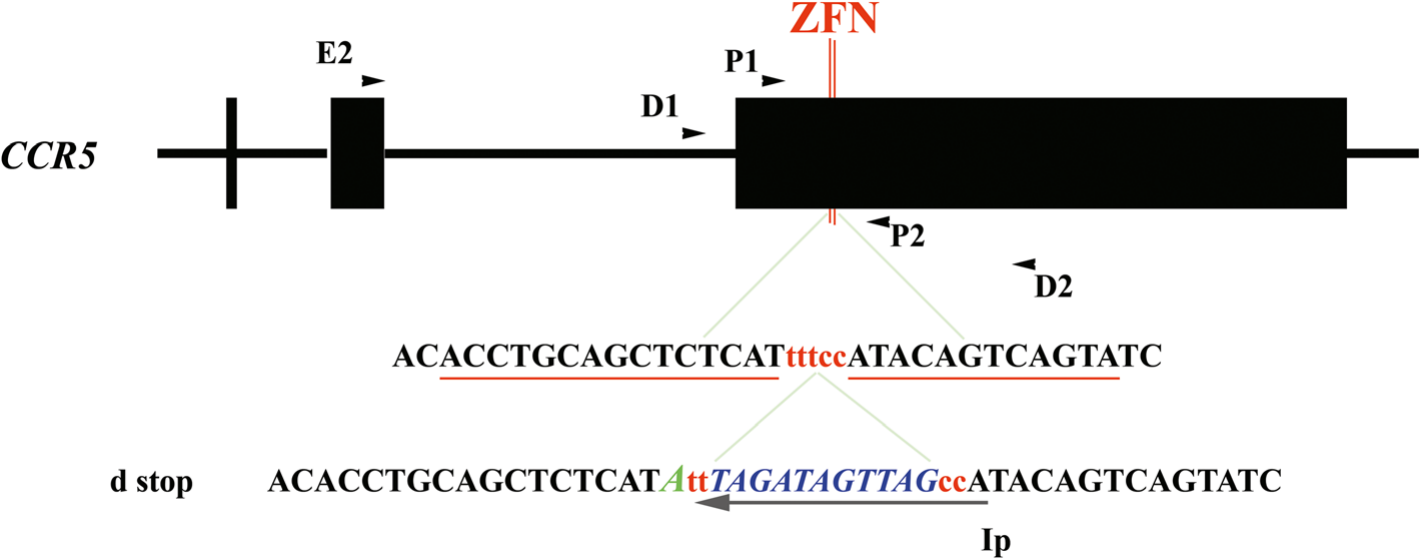
**Primers map and donor plasmid generation.** Illustration of the *CCR5* locus and primer locations. *Upper panel*: ZFN-targeted DNA sequence (underlined) and the interspace. *Lower panel*: DNA sequences of the d-plasmid around the ZFN site and the insertion-specific primer (IP); a silent T-A mutation adjacent to the left ZFN-targeted site was intentionally selected for confirmation and marking of the vicinity of integration between the ZFN-binding sites.

cDNA of human *CCR5* (1791 bp), from −733 bp upstream of the left-hand ZFN-binding site to 1038 bp downstream of the right-hand ZFN-binding site, was amplified from genomic DNA of peripheral blood using the primers D1 (5′-GTGGACAGGGAAGCTAGCAG-3′) and D2 (5′-CCATACCTTGGAGGGGAAAT-3′). The polymerase chain reaction (PCR) products were ligated into a TA cloning vector (RBC TA Cloning Vector Kit, RBC Bioscience; Taipei, Taiwan). Next, the ligated vectors were transformed into *E. coli* competent cells (Solo Pack Gold; Agilent Technologies; Santa Clara, CA, USA) and subjected to sequencing analysis. We designed the universal stop codon “TAGATAGTTAG” and inserted it between two ZFN-binding sites by PCR-induced mutagenesis (Agilent Technologies). The insertion was confirmed by DNA sequencing and the plasmid was designated as d-stop plasmids (Figure 1).

### Editing of *CCR5* in MSCs with ZFN

*CCR5*-specific ZFN mRNA was purchased from Sigma Aldrich (St. Louis, MO, USA). The ZFN was designed to target the third exon of *CCR5*, as previously described [13]. In some experiments, we synthesized the pair of ZFN mRNAs from a plasmid by using the Massagemax T7 mRNA transcription and Poly (A) polymerase tailing kit (Epicentre; Madison, WI, USA). The ZFN mRNA pair and d-plasmid were transfected to MSCs with Nucleofector (Neon; Invitrogen; Carlsbad, CA, USA). Cells were cultured for 5 days after transfection, and were then trypsinized and replated into 96-well plates at a density of 1 cell/well or 6 cells/well. Cells in 96-well plates were examined on a daily basis. After growing to a 70% confluent monolayer, MSCs from the single-cell or six-cell wells were trypsinized and transferred to two 3-cm Petri dishes.

### Nested PCR screening for HDR-mediated stop codon insertion

Genomic DNA was extracted from confluent 3-cm culture plates that originated from single-cell or six-cell clones with a genomic DNA extraction kit (RBC Bioscience). The extracted DNA was then subjected to nested PCR. The first round of amplification was carried out with primers E2 (5′-CTGAGACATCCGTTCCCCTA-3′; −1744 bp upstream of D1) and P2 (5′-TGTAGGGAGCCCAGAAGAGA-3′). Once the first round was complete, the second round of PCR was established with an insertion-specific primer (IP) (5′-TGGCTAACTATCTAAA-3′) and P1 (5′-TTAAAAGCCAGGACGGTCAC-3′) (Figure 1). The PCR products were electrophoresed on 1.5% agarose gel (Pronadisa; Madrid, Spain) and visualized under ultraviolet light. To assess the amount of stop codon integration in the expandable six-cell clones, the genomic sequence of *CCR5* was amplified using the P1/P2 primer pair. PCR products were ligated to TA cloning vector (RCB Bioscience) and transformed into *E. coli*. The bacteria colonies were picked up and amplified with the P1, P2, and IP primers. The ratios of double 152-bp and 346 + 11 (357)-bp bands, which represented of stop codon insertion per successful transformation, which represented by total of single 346-bp and double 152-bp,357-bp bands, were then calculated.

### Genomic gene sequencing

To confirm the presence of the inserted sequence, PCR products of positively screened single- and six-cell clones were ligated and transformed into *E. coli* competent cells. Plasmid DNA from each colony was extracted and subjected to direct sequencing or reamplification with the P1/P2 primer pair before sequencing. The sequencing was carried out by Macrogen Company (Seoul, Korea).

### Hematopoietic progenitor conversion of gene-edited MSC clones

A retroviral vector encoding human Octamer-binding transcription factor 4 (Oct4) was purchased from Cell Biolabs (San Diego, CA, USA). The vector was transfection to Plat-A packaging cells with X-fect reagent (Clontech; Mountain View, CA, USA) for retrovirus construction. Forty-eight hours after vector transfection, the virions were harvested and concentrated with Retro-X Concentrator (Clontech). Direct conversion of gene-edited MSCs to hematopoietic progenitor cells was carried out in five independent experiment (2 mutated six-cell clones and 3 native MSCs).Ten thousand of native MSCs or MSCs from *CCR5*-mutated MSC clones were plated on Matrigel-coated flasks and cultured in DMEM/F-12 supplemented with 10% fetal bovine serum (FBS; Gibco; Grand Island, NY, USA). Twenty-four hours after plating, cells were infected with a retrovirus expressing human *Oct 4* cDNA by polybrene (Sigma). One day after infection, culture media containing the virus were replaced with reprogramming media (RM) (DMEM/F-12 supplemented with 10% FBS and 100 units/ml penicillin G-streptomycin, 0.1 mM β-mercaptoethanol,16 ng/ml basic fibroblast growth factor [R&D Systems], 30 ng/ml insulin-like growth factor-2, 300 ng/ml Flt3, and 300 ng/ml stem cell factor). Mutated and non-mutated MSCs that overexpressed human Oct4 and the control MSCs that were maintained in RM were analyzed by flow cytometry (FACS Calibur; BD Biosciences) on day 14.

## Results

### Isolation and characterization of MSCs

**Figure 2:**
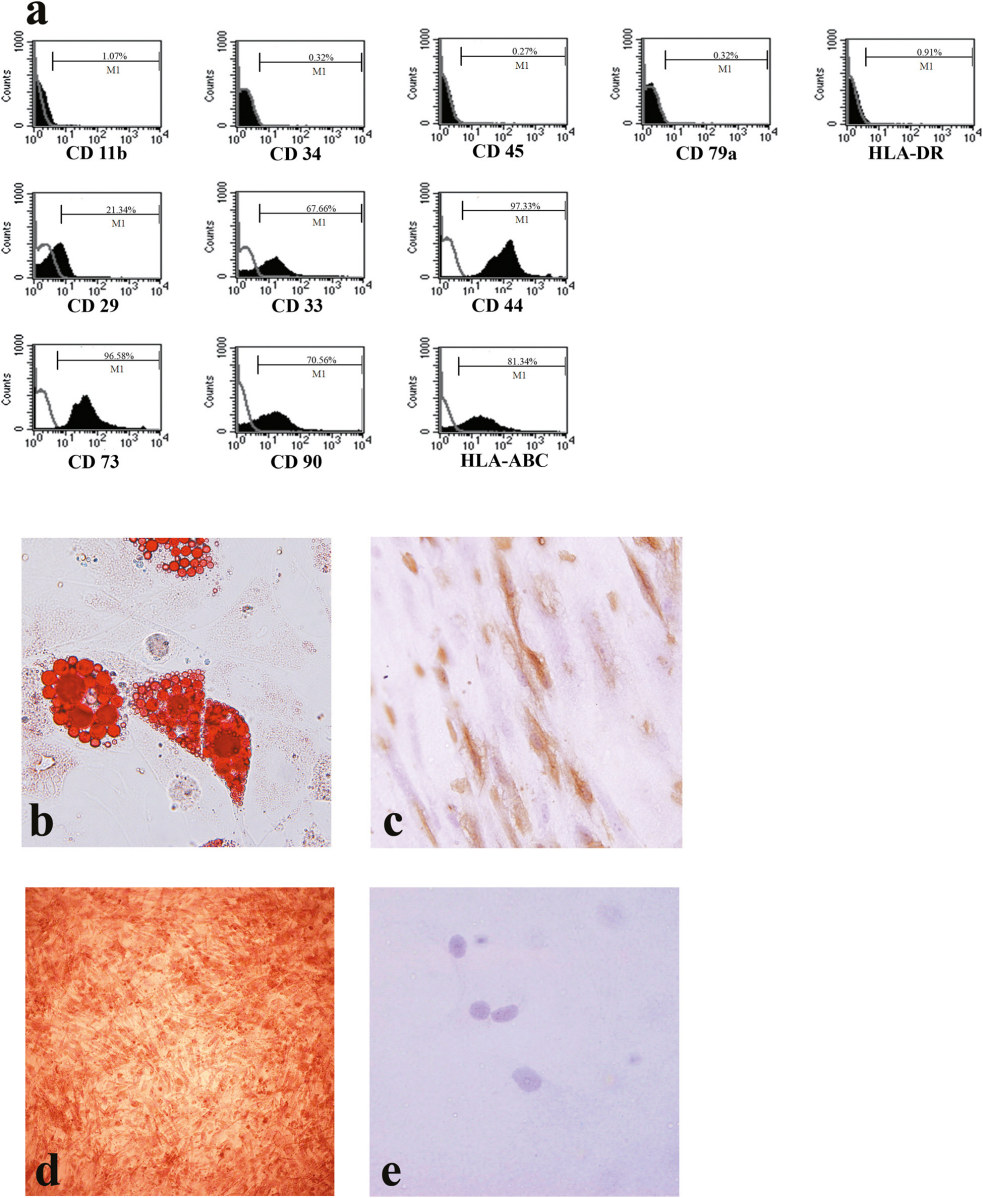
**Basic properties of MSCs. a)** Flow cytometry analysis of MSCs. **b)** Adipocyte differentiation as shown by positive Oil Red O lipid accumulation in the cytoplasm. **c)** MSCs expressed osteocalcin in the cytoplasm after osteogenic differentiation was induced. **d)** Calcium deposition in MSCs after osteogenic differentiation demonstrated by Alizarin Red staining. **e)** Immunohistochemistry of CCR5 showed that the MSCs were negative for CCR5 expression.

We isolated MSCs from leftover bone marrow aspiration specimens that were used for hematologic diagnosis of four donors (Table 1). Flow cytometry analysis showed that these cells were positive for mesenchymal surface markers such as CD29, CD44, CD73, CD90, and HLA-ABC and were negative for CD11b, CD34, D45, CD79a, and HLA-DR (Figure 2a.). These cells were able to differentiate into adipocyte and osteocyte lineages, as shown in Figure 2b,c and d. Immunohistochemistry suggested that these cells did not express CCR5 (Figure 2e).

**Table 1:** **Patient details**

**Patient**	**Clinical setting**	**Single-cell clone**	**Six-cells clone**
1	Male, HIV+, with thrombocytopenia, ARVs+	1	Not done
2	Female, HIV+, with pancytopenia, ARVs+	0	Not done
3	Female, ERSD, with erythropoietin unresponsive anemia, HIV-	1	1
4	Male, Alcoholic cirrhosis with thrombocytopenia, HIV+, ARV-	Not done	3

### Direct generation of isogenic ZFN-mediated stop codon *CCR5* clones

**Figure 3:**
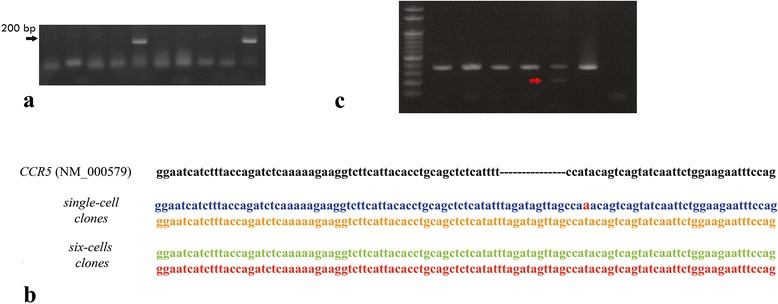
**PCR screening and DNA sequencing. a)** Nested PCR showing the positive clones in lane 5 and 10. **b)** DNA sequence of isogenic single-cell and six-cell clones. (Note the A → T mutation in one of the isogenic clones). **c)** PCR products of *CCR5* from picked-up colonies; the double bands indicated the existence of an HR-mediated mutation (arrow; indicated the lower band), while the single band indicated the presence of the *CCR5* sequence in the bacteria colonies. The ratios of double/total of single and double band were 12.2%and 13.3%, suggesting that both six-cell clones initially carried only one mutation of 12 alleles.

A total of 2 × 10^6^ MSCs (passage 3–5) were transfected with mRNA encoding ZFNs targeting the *CCR5* gene along with the d-stop plasmid by nucleoporation. Five days after gene transfection, MSCs were trypsinized and replated in 96-well plates at a density of one cell per well. A total of 4320 single-cell cultures were established (3 donors, 8 independent experiments). The cultured cells gradually propagated and expanded. By the 8^th^ week, only 88 clones that originated from a single cell successfully expanded to reach an 80% confluent monolayer in two 3-cm dishes. Nested PCR screening revealed that 2 of the 88 clones carried the insertion sequence in the *CCR5* locus (Figure 3a). Genomic sequencing confirmed that both isogenic clones contained the single-allele HDR-mediated stop codon insertion (Figure 3b). However, neither of the inserted clones could propagate beyond two passages in 25-ml flasks (≈0.8 million-fold). This might be due to the expansion limit of MSCs [31].

### Generation of ZFN-mediated stop codon *CCR5* six-cell clones

Previous studies indicated that the proliferation of MSCs depends on the initial seeding density [34]. To circumvent the limited expansion observed in single-cell clones and to enhance ex *vivo* enrichment, we, therefore, increased the initial density to six cells/well. From two donors, we set up three independent gene-editing experiments in an attempt to generate a total of 986 six-cell clones. Of these, 42 six-cell clones were generated within four weeks and were screened for stop codon insertion. Nested PCR screening showed that four clones had the inserted stop codon in the *CCR5* locus. Of these, two clones were expandable in a 25-ml flask. Unlike the single-cell clones, these six-cell clones were able to further expand in the 25-ml flask beyond five passages (at a 1:2 split) before the proliferation rate decreased (≈1 million-fold). The insertion was stably detected at all five passages. The ratios of double PCR products per transformation success in the two clones at 3^rd^ passage (≈7 weeks after ZFN editing) were 12.2% and 13.3%, respectively (Figure 3c). Although, our estimation method of gene integration might be confounded if the donor plasmid were present in the established clones. However, after 7 weeks of expansion culture, it was unlikely that TA cloning vector still retained and contributed to 12-13%of PCR products. Taken together, the result suggested that both clones initially had only one cell with a single allele of HDR-mediated gene insertion.

### Conversion of native MSCs and edited MSC clones to CD45+/CD34+ cells

**Figure 4:**
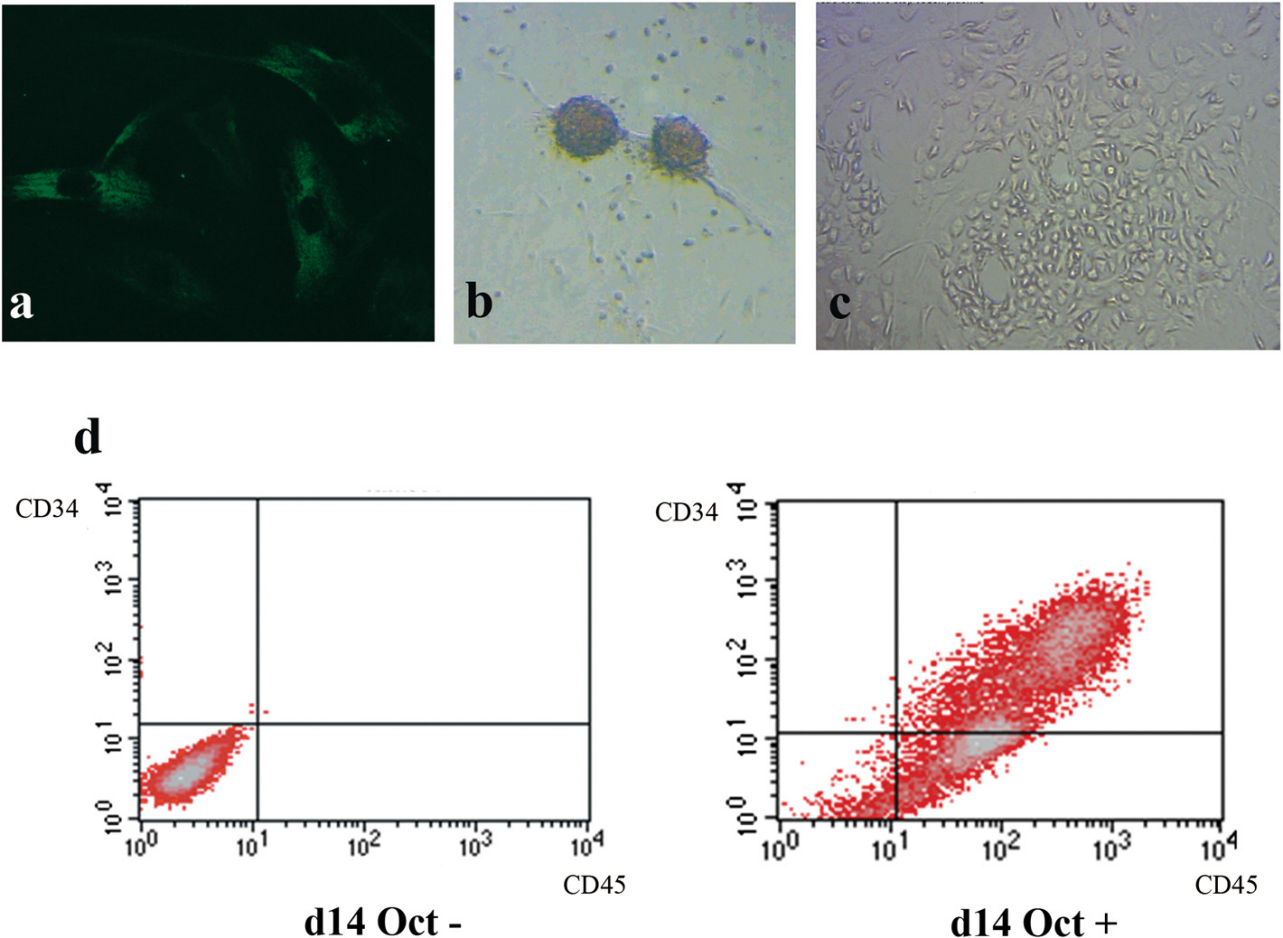
**Direct cell conversion by Oct4. a)** Immunofluorescence of Oct4 at 24 h after infection. **b)** Representative picture of the appearance of the mutated colonies at day 7–10 after Oct4 infection. **c)** Small cells that rapidly expanded from the emerging colonies. **d)** Representative image of flow cytometry of a six-cell-mutated clone at day 14, showing the emergence of CD34+ and CD45+ cells in Oct4 transduced cells as compared to the six-cell-mutated control.

In order to support the practical utility of the mutated *CCR5* MSCs, we next converted the mutated cells to CD34+ cells by infection with a retrovirus encoding Oct4. Immunofluorescence analysis at 24 h after infection demonstrated that approximately 60% of the MSCs expressed Oct4, mostly in the cytoplasm (Figure 4a). Around day 7–10 after infection, small colonies of cells began to appear before rapidly proliferating and expanding (Figure 4b). Cells from the expanding colonies were smaller and rounder than normal MSCs (Figure 4c). These round cells had a tendency to float rather than adhere to the Matrigel-coated plate. The proportion of round cells peaked at day 14 before decreasing at day 21. Flow cytometry analysis at day 14 post-infection showed an increase in CD34+ cells (Figure 4d) in Oct4-transfected MSCs as compared to the nontransfected clones. Cells expressing the pan-leukocyte marker CD45 also increased in parallel with CD34+ cells; at day 14, approximately 33.6% of the cells showed double-positive expression of CD34 and CD45.

## Discussion

The progress related to the use of ZFN-edited *CCR5* for potential HIV treatment has been very rapid. A number of studies have confirmed the reproducible success of genome editing of *CCR5* loci in various cell types and with the use of different methods [10-14,19,25,35-42]. Currently, ZFN editing of patients’ own CD4 lymphocytes has moved into phase I trials, and new methods for incorporating this technique to large scales are currently under investigation [38,39]. Since genome editing occurs individually in each cell, the key factors for the success of this technique with respect to clinical applications are the editing efficacy and the expansion ability of the edited progeny cells. Recently, the InDel mutation of CD34+ cells has been reported [14,40,41]. Unlike CD4 lymphocytes, which are terminally differentiated cells and thus have limited ability for *in vivo* proliferation, CD34+ HSCs can regenerate entire bone marrow populations. Therefore, their potential to function as an autologous BMT source for HIV treatment seems very promising. It is known that the success of BMT depends on the CD34+ cell dose [41]; hence, generating a critical number of edited CD34 cells is a key factor to the success of this technique for treatment. Unfortunately, the *in vitro* expansion of CD34 cells is relatively restricted, and thus would not be suitable for clonal generation.

The advantages of stem cells are their unlimited self-renewal and broad ability of differentiation. In the present study, we took advantage of these properties of bone marrow-derived MSCs to generate CD34+ clones that carried an HDR-mediated stop codon insertion of *CCR5*, an important co-receptor for HIV to enter the cell, without using selectable markers. While this work was in progress, Yao’s group employed ZFN to integrate exogenous green fluorescent protein into *CCR5* loci of induced pluripotent stem cell (iPSC) and embryonic stem cell lines [26]. Using PCR screening, the authors estimated that the rate of HDR-mediated gene integration was remarkably high in both cell lines. Of particular interest, the edited cells could further differentiate into CD34 cells and hematologic lineage cells. Theoretically, Yao’s approach can produce patient-specific isogenic CD34 cells for therapeutic purposes; however, expanding HSCs from iPSCs remains a technically difficult task.

In our work, with mRNA-based ZFN-mediated HDR, we could obtain only 6 expandable cells out of 10,236 attempted cells that carried one allele of gene insertion; therefore, the yield of our experimental conditions should not be lower than 0.059%. Despite the fact that this yield is lower than that obtained with other methods of ZFN/donor delivery, we clearly showed that the proliferation of MSCs is high enough to overcome the low initial yield. It is also possible that employing either drug or marker selection might increase the number of positive clones; however, we consider that the use of a direct clone selection method is more straightforward and safe for future clinical applications. The recent emergence of new genome-editing tools, transcription activator-like effector nucleases (TALEN) and clustered regularly interspaced short palindromic repeats (CRISPR), is of interest in this respect [37,42]. Several reports have shown that the efficiency of genome disruption with both methods was higher than that of ZFN, which might help to improve the overall yield.

We also evaluated the feasibility of using the mutated MSC clones for HIV treatment by converting these cells to CD34+ cells. In the pioneer work on direct conversion of fibroblasts to HSCs, the investigators found that the number of CD45+/CD34+ cells had significantly increased 3 weeks after overexpression of Oct4 was induced [30]. Recent work from the same group further clarified that both Oct4 and culture media, RM, were crucial for the conversion and maintenance of the HSC phenotype [43]. In our work, both native and ZFN-edited MSCs expressed CD34 after induction with Oct4 and culture in RM. Of particular note, a significant number of CD34+ cells showed co-expression of CD45 antigens; both antigens are important surface markers of the hematologic lineage and are always negative in MSCs, indicating successful lineage conversion. However, it should also be noted that the retrovirus mediating Oct4 is not safe for clinical use and single allele of CCR5 disruption is insufficient to inhibit viral entering. Nonetheless, these results demonstrate the great potential of this approach in future HIV treatment.

## Conclusion

We here reported the novel approach on generation of patients own CD34 cells from high fidelity ZFN-mediated HDR MSC clones. We believe that our approach will be beneficial in future HIV treatment.

## Abbreviations

ARV: Antiretroviral drug
BMT: Bone marrow transplantation
CCR5: Chemokine receptor 5
DMEM: Dulbecco’s modified Eagle’s medium
DSB: Double-strand break
HDR: Homology-directed repair
HIV: Human immunodeficiency virus
HLA: Human leukocyte antigen
HSC: Hematopoietic stem cell
MSC: Mesenchymal stem cell
NHEJ: Non-homologous end-joining
PBS: Phosphate-buffered saline
PCR: Polymerase chain reaction
Oct4: Octamer-binding transcription factor 4
ZFN: Zinc finger nuclease
